# Challenges and Contradictions of Metal Nano-Particle Applications for Radio-Sensitivity Enhancement in Cancer Therapy

**DOI:** 10.3390/ijms20030588

**Published:** 2019-01-30

**Authors:** Eva Pagáčová, Lenka Štefančíková, Franz Schmidt-Kaler, Georg Hildenbrand, Tomáš Vičar, Daniel Depeš, Jin-Ho Lee, Felix Bestvater, Sandrine Lacombe, Erika Porcel, Stéphane Roux, Frederik Wenz, Olga Kopečná, Iva Falková, Michael Hausmann, Martin Falk

**Affiliations:** 1 Czech Academy of Sciences, Institute of Biophysics, v.v.i., Kralovopolska 135, 612 65 Brno, Czech Republic; pagacova@ibp.cz (E.P.); StefancikovaL@seznam.cz (L.S.); depesd26@gmail.com (D.D.); kopecna@ibp.cz (O.K.); ivafalk@ibp.cz (I.F.); 2 Institute des Sciences Moléculaires d’Orsay (ISMO), Université Paris Saclay, Université Paris Sud, CNRS, 91405 Orsay Cedex, France; sandrine.lacombe@u-psud.fr (S.L.); erika.porcel@u-psud.fr (E.P.); 3 Kirchhoff-Institute for Physics, University of Heidelberg, Im Neuenheimer Feld 227, 69120 Heidelberg, Germany; franzschmidtkaler@web.de (F.S.-K.); hilden@kip.uni-heidelberg.de (G.H.); jin-ho.lee@kip.uni-heidelberg.de (J.-H.L.); 4 Department of Radiation Oncology, Universitätsmedizin Mannheim, Medical Faculty Mannheim, Heidelberg University, 68159 Mannheim, Germany; Frederik.Wenz@medma.uni-heidelberg.de; 5 Brno University of Technology, Department of Biomedical Engineering, Technická 3082/12, 61600 Brno, Czech Republic; tomasvicar@gmail.com; 6 German Cancer Research Center (DKFZ), Im Neuenheimer Feld 280, 69120 Heidelberg, Germany; f.bestvater@dkfz.de; 7 Institute UTINAM, UMR CNRS 6213-Université de Bourgogne Franche-Comté, 25020 Besançon Cedex, France; stephane.roux@univ-fcomte.fr

**Keywords:** metal nanoparticles, cancer radiotherapy, tumor cell radiosensitization, DNA damage, DNA repair, DNA double strand breaks (DSBs), super-resolution microscopy, single-molecule localization microscopy (SMLM), DNA repair foci, damage to lysosomes

## Abstract

From the very beginnings of radiotherapy, a crucial question persists with how to target the radiation effectiveness into the tumor while preserving surrounding tissues as undamaged as possible. One promising approach is to selectively pre-sensitize tumor cells by metallic nanoparticles. However, though the “physics” behind nanoparticle-mediated radio-interaction has been well elaborated, practical applications in medicine remain challenging and often disappointing because of limited knowledge on biological mechanisms leading to cell damage enhancement and eventually cell death. In the present study, we analyzed the influence of different nanoparticle materials (platinum (Pt), and gold (Au)), cancer cell types (HeLa, U87, and SKBr3), and doses (up to 4 Gy) of low-Linear Energy Transfer (LET) ionizing radiation (γ- and X-rays) on the extent, complexity and reparability of radiation-induced γH2AX + 53BP1 foci, the markers of double stand breaks (DSBs). Firstly, we sensitively compared the focus presence in nuclei during a long period of time post-irradiation (24 h) in spatially (three-dimensionally, 3D) fixed cells incubated and non-incubated with Pt nanoparticles by means of high-resolution immunofluorescence confocal microscopy. The data were compared with our preliminary results obtained for Au nanoparticles and recently published results for gadolinium (Gd) nanoparticles of approximately the same size (2–3 nm). Next, we introduced a novel super-resolution approach—single molecule localization microscopy (SMLM)—to study the internal structure of the repair foci. In these experiments, 10 nm Au nanoparticles were used that could be also visualized by SMLM. Altogether, the data show that different nanoparticles may or may not enhance radiation damage to DNA, so multi-parameter effects have to be considered to better interpret the radiosensitization. Based on these findings, we discussed on conclusions and contradictions related to the effectiveness and presumptive mechanisms of the cell radiosensitization by nanoparticles. We also demonstrate that SMLM offers new perspectives to study internal structures of repair foci with the goal to better evaluate potential differences in DNA damage patterns.

## 1. Introduction

More than a half of all cancer patients are currently treated with radiotherapy [1] that, together with chemotherapy, still represents the most efficient curative approach for many cancer types. The therapeutic window of radiotherapy (and chemotherapy) [2] is based on different capacities of normal and cancer cells to repair DNA damage. Because of defects in cell cycle checkpoints and/or repair pathways [3], cancer cells more or less suffer from genomic instability and are more susceptible than normal cells to DNA-damaging agents. Some tumors are highly radioresistant though, making them difficult to eradicate while preserving the surrounding normal tissues undestroyed [4,5,6]. A crucial part of cancer treatment development therefore concerns a question of how to deliver the radiation effectiveness into the tumor while preserving the normal surrounding tissues as much as possible. This issue becomes of fundamental importance for radioresistant tumors and/or tumors located in close proximity of vital organs or structures. An illustrative example could be the most aggressive and radioresistant tumor [7] starting in the brain—glioblastoma—leading us to select U87 glioblastoma cells as a model in the present study. HeLa cells, an often used model in bio-medical research, were included in the present study as a different cancer cell type for their lower radioresistance and different origin. SkBr3 cells [8] were involved as a model for breast cancer with Her2/neu up-regulation, on which the radiation effects are studied in combination with antibody and/or chemo-treatment [9].

Several promising strategies are continuously being developed to improve radiotherapy. For instance, spatial dose fractionation, time dose fractionation, micro/mini-beam irradiation, heavy-ion irradiation [10,11,12,13,14,15,16,17], and application of normal cell radio-protectants [18,19] and/or tumor cell radiosensitizers [20] could already be used in practice and eventually combined. One of the radiosensitizing approaches proposed is to selectively potentiate radiation toxicity for tumor cells by metal nanoparticles [21,22,23,24,25,26,27,28]. Due to their high electron content and photoelectric absorption cross-section, metal (high atomic number = high-Z material) nanoparticles emit showers of secondary electrons upon irradiation [29,30]. Launched electrons then generate clouds of high ionization densities, capable of enhancing radiation-induced cell damage and death rates [31].

The cell nucleus and DNA located therein are sensitive to many stressors [32,33,34,35,36] and can be highly damaged with relatively low doses of ionizing radiation [37,38]. As deleterious effects of ionizing radiation on (cancer) cells are mostly mediated through fragmentation of nuclear chromatin by inserting double strand breaks (DSBs) into the DNA molecule [39], nanoparticle radiosensitizing effects have primarily been ascribed to an increased number and/or complexity of DSBs generated by radiation in presence of nanoparticles [23]. Clustered (complex) DSBs can only be repaired with difficulty [16,40,41] and were recognized as the main factor responsible for the superior radiobiological efficiency (RBE) of densely ionizing radiations. Hence, according to this hypothesis, at a given absorbed dose and irradiation parameters, nanoparticles boost cell killing by locally amplifying the dose [42] and, in turn, DNA damage. Indeed, increased numbers relative to untreated samples of single-stranded breaks (SSBs) and DSBs were measured in DNA irradiated in the solution with various metal nanoparticles [43]. Since nanoparticles are preferentially internalized and accumulated by cancer cells, even passively due to mechanisms collectively known as the so-called Enhanced Permeability and Retention (EPR) effect, these enhancement effects of radiotherapy could be selectively targeted to tumors [44,45,46,47,48,49,50]. Moreover, some nanoparticles exert dual multiple benefits in cancer treatment at the same time—they can be used as contrast agents in theranostics [51] and/or vehicles for delivery of various chemotherapeutics or biological treatment compounds to the tumor. Nanoparticles can be also functionalized (surface material modification, attached antibodies, size, shape, etc.) to better identify and infiltrate the tumor [23,48]. Moreover, nanoparticles can be used as imaging tags especially where photo-bleaching has to be avoided [52,53].

Aforementioned physical predictions on the mechanism of nanoparticle-mediated radiosensitization were confirmed experimentally [51]. As already noticed, isolated DNA showed increased fragmentation after being irradiated in presence of various nanoparticles [43]. In other experiments, nanoparticles also increased cell dying when being added to cell cultures prior to irradiation [21,43,54]. Nevertheless, it is in fact not so easy to explain the nanoparticle-mediated cell radiosensitization, despite seemingly ideal correspondence between the theoretical predications and experimental results. The Achilles’ heel of the current “mainstream” hypothesis followed from in situ/in vivo experiments showing that nanoparticles, even those of very small dimensions (e.g., of 2–3 nm in diameter, as used in this work), penetrate the cells but not the cell nucleus [21,23,53,55] unless they are specifically modified for this purpose [42]. Nanoparticles of different materials and sizes, entering the cells by pinocytosis (reviewed in [56]), thus remain retained inside the cytoplasm, where they accumulate especially in endoplasmic vesicles (endosomes) and lysosomes [21,55,57]. Under some circumstances, nanoparticles may co-localize preferentially with the endoplasmic reticulum (ER) [23,58] and Golgi apparatus (reviewed in [56]). Interestingly, mitochondria, the only cytoplasmic organelles in human cells that contain their own DNA, do not represent a primary target for nanoparticles, though some nanoparticles can also be targeted to these structures (reviewed in [56]). These findings put into play a plethora of various cellular processes potentially participating in nanoparticle-mediated tumor cell radiosensitization. It is therefore possible that different nanoparticles do not share a common mode of action, both in terms of the type of cell damage and its underlying mechanism (reviewed in [59]).

In the present work, we analyzed for different metal nanoparticles, whether their extra-nuclear [21,22,23,55] presence in cells can, by itself or upon cell irradiation, enhance damage of the nuclear DNA. In addition, we followed in detail nanoparticle effects on the kinetics and efficiency of DNA repair in cells exposed to low-Linear Energy Transfer (LET) ionizing radiation (γ- and X-rays). We used high-resolution immunofluorescence confocal microscopy (ICM) and single molecule localization microscopy (SMLM) [38,60] to quantify γH2AX/53BP1 DSB repair foci formation [61] and disassembly during a long period of time post-irradiation (PI) in cells exposed to different doses of γ/X-rays after being or being not incubated with platinum nanoparticles (Pt-NPs). On the basis of these data, preliminary data for gold nanoparticles (Au-NPs), and our earlier data for gadolinium nanoparticles (Gd-NPs) [21], we discussed here on what is known about metal nanoparticle effects on cells and potential mechanisms of nanoparticle-mediated radiosensitization.

In general, the aim of the following study was to verify whether cell radiosensitization by metal nanoparticles is correlated with escalation of DNA damage and/or affection of DNA damage repair capacity. The results should contribute to a better understanding of the mechanism by which various nanoparticles (different materials and sizes) radiosensitize cells with future attempt to rationally design therapeutically more efficient nanoparticles.

## 2. Results

### 2.1. Experimental Conditions and Approaches

We explored how platinum (Pt) and gold (Au) nanoparticles influence DNA DSB induction and repair in three different cancer cell types, U87 glioblastoma cells, HeLa cervix cancer cells and SkBr3 breast cancer cells, exposed to γ-(^137^Cs) or X-radiation. U87 glioblastoma cells were selected for their high resistance to radiotherapy. HeLa cells, showing relatively lower resistance to radiation, were then involved into the study to explore how tumor cell types of different radiosensitivities and origins respond to nanoparticle uptake and nanoparticle uptake followed by irradiation. The SkBr3 model is known to be more radioresistant in comparison to HeLa cells and was especially taken for super-resolution localization microscopy. The mechanism and kinetics of nanoparticle internalization were in detail evaluated in our previous studies with Gd-NPs (3 ± 1 nm) [21,55] and Au-NPs (10 nm) [53] of a comparable size to Pt-NPs (2.6 nm) and Au-NPs (2.4 nm) used in the present study. We showed that these ultrafine Gd-NPs as well as the larger Au-NPs efficiently penetrate into the cell cytoplasm but remain restricted from the cell nucleus. Even short (2 h) incubation with nanoparticles was proved to be sufficient to ensure their internalization and cell radiosensitization upon irradiation with γ/X-rays [53]. Therefore, we used the compatible conditions also here, though lower concentration and longer incubation period (0.5 mM/6 h) were preferred to ensure sufficient cell accumulation but minimize the potential cytotoxicity.

**Figure 1:**
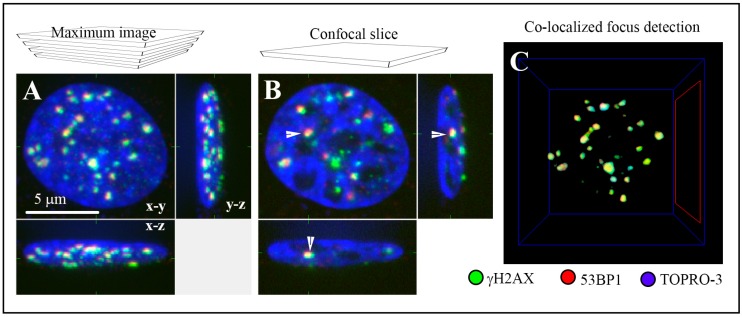
The ability of immunofluorescence confocal microscopy to quantify DSBs (Double Strand Breaks) in cells incubated with nanoparticles or incubated with nanoparticles and consecutively irradiated. DSBs were quantified by the means of immunofluorescence detection of co-localized γH2AX (green) and 53BP1 (red) repair foci, the DSB markers. The nucleus of an illustrative U87 cell exposed to 2 Gy of γ-rays and spatially (three-dimensionally = 3D) fixed at 2 h post-irradiation (PI) is shown as: (**A**) a maximum intensity projection of 40 confocal slices (0.3 µm thick; “maximum image”) or (**B**) a single confocal slice (0.3 µm thick) intersecting the indicated (white arrow) γH2AX/53BP1 focus. Images are displayed in all three (in the x-y, x-z and y-z) planes, and chromatin is counterstained with TO-PRO-3 (artificially blue). (**C**) An example of computational detection of co-localized (yellow) γH2AX (green) and 53BP1 (red) repair foci in 3D space (Aquarium Software).

Modern, top-tech microscopy approaches were used in the present study to analyze DNA damage and repair with high precision. ICM allowed for quantification of DSBs during a long PI time period [16] (Figure 1). When co-localized γH2AX and 53BP1 repair foci are used as DSB markers, the sensitivity of the method is clearly superior over other modern methods, including fluorescence COMET assay (single cell gel electrophoresis) on single cells [20]. Newly developed SMLM, used here for super-resolution ultra-structural analyses of repair foci [61], offers even better resolution (up to 10–20 nm) and sensitivity than ICM. Nevertheless, since higher numbers of cells can be currently analyzed by ICM, we took advantage of this method to determine the extent of DSB induction and DSB repair kinetics in statistically relevant numbers of spatially (three-dimensionally = 3D) fixed cells, still with a very high credibility and fidelity of analysis. To further increase the credibility of our study, we scored the repair foci both manually and automatically. This also allowed us to compare the positives and negatives of both approaches and to determine the influence of γH2AX/53BP1 focus scoring method on the results. 

### 2.2. Pt-NP and Au-NP Short-Term Genotoxicity—the Effect on Nuclear DNA in Non-Irradiated Cells

**Figure 2:**
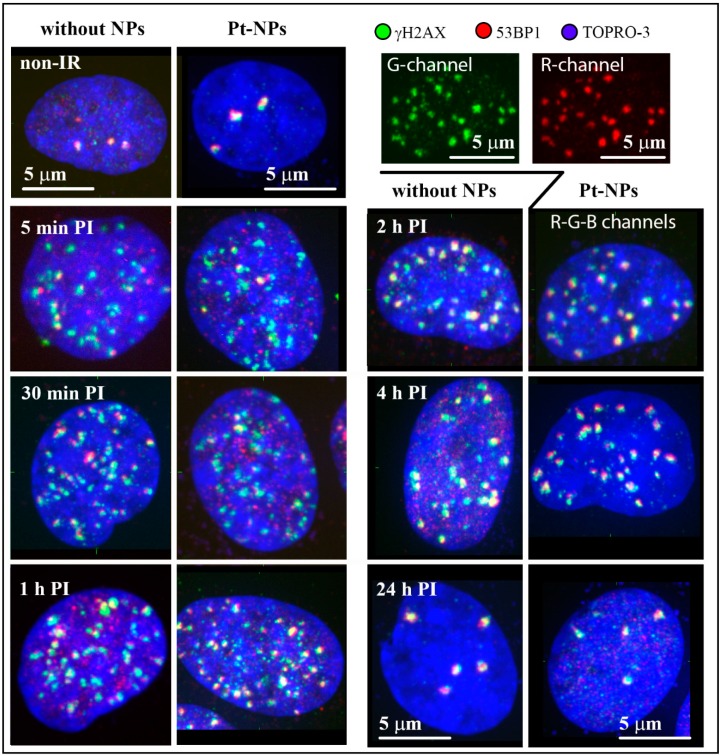
γH2AX/53BP1 foci (DSB) formation and repair kinetics in U87 cells incubated or not incubated with 2.6 nm platinum nanoparticles (Pt-NPs; 0.5 mM for 6 h) and consequently irradiated with 4 Gy of γ-rays. Maximum images (see Figure 1) are displayed for representative nuclei of cells that were spatially (3D) fixed in the indicated periods of time PI. For the nucleus fixed at 2 h PI, γH2AX foci (inserted G-channel panel) and 53BP1 foci (inserted R-channel panel) are also shown separately to demonstrate their mutual co-localization. γH2AX (green), 53BP1 (red), and chromatin counterstained with TO-PRO-3 (artificially blue). None-IR figures correspond to non-irradiated cells.

**Figure 3:**
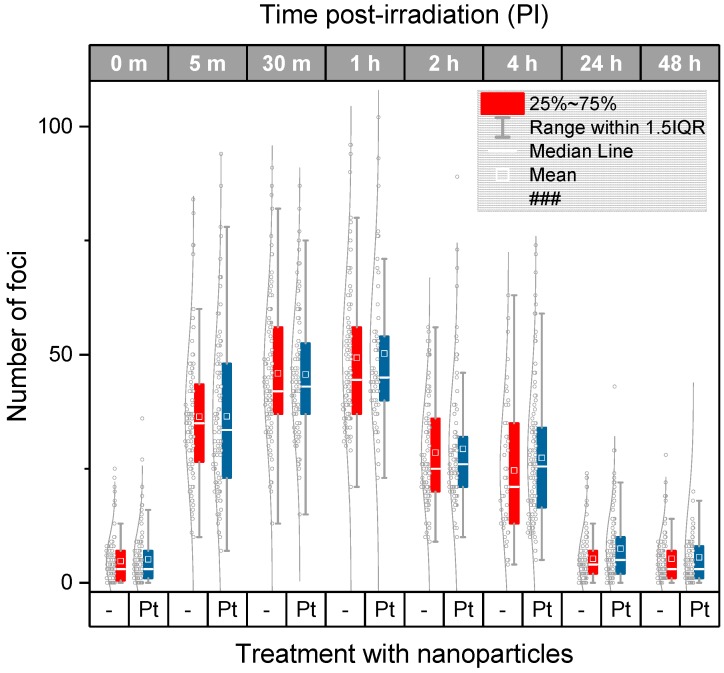
Manual analysis of the extent of γH2AX+53BP1 focus (DSB) induction and repair kinetics in U87 glioblastoma cells irradiated with 4 Gy of γ-rays compared with cells treated (0.5 mM for 6 h) and not treated prior to irradiation with 2.6 nm platinum nanoparticles (Pt-NPs). The average and median numbers of co-localized γH2AX + 53BP1 repair foci (i.e., DSBs) per nucleus are shown for different periods of time PI, together with the focus number distributions in each cell population. The boxes include 50% of the values (25th to 75th percentile) centered on the median (the horizontal line through the box). The mean values are represented by the squares within the boxes. The outliers were identified according to the 1.5*IQR method (IQR = interquartile range). Pt—samples treated with platinum nanoparticles, m—the period of time after irradiation in minutes, 0 m—non-irradiated samples.

**Figure 4:**
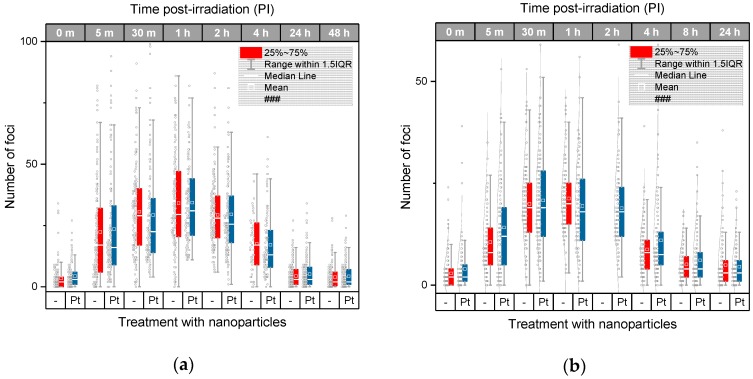
Software analysis of the extent of γH2AX+53BP1 focus (DSB) induction and repair kinetics in U87 glioblastoma cells irradiated with 4 Gy (**a**) or 2 Gy (**b**) of γ-rays compared with cells treated (0.5 mM for 6 h) or not treated prior to irradiation with 2.6 nm platinum nanoparticles (Pt-NPs). The average and median numbers of co-localized γH2AX + 53BP1 repair foci (i.e., DSBs) per nucleus are shown for different periods of time PI, together with the focus number distributions in each cell population. The boxes include 50% of the values (25th to 75th percentile) centered on the median (the horizontal line through the box). The mean values are represented by the squares within the boxes. The outliers were identified according to the 1.5*IQR method (IQR = interquartile range). Pt—samples treated with platinum nanoparticles, m—the period of time after irradiation in minutes, 0 m—non-irradiated samples.

**Figure 5:**
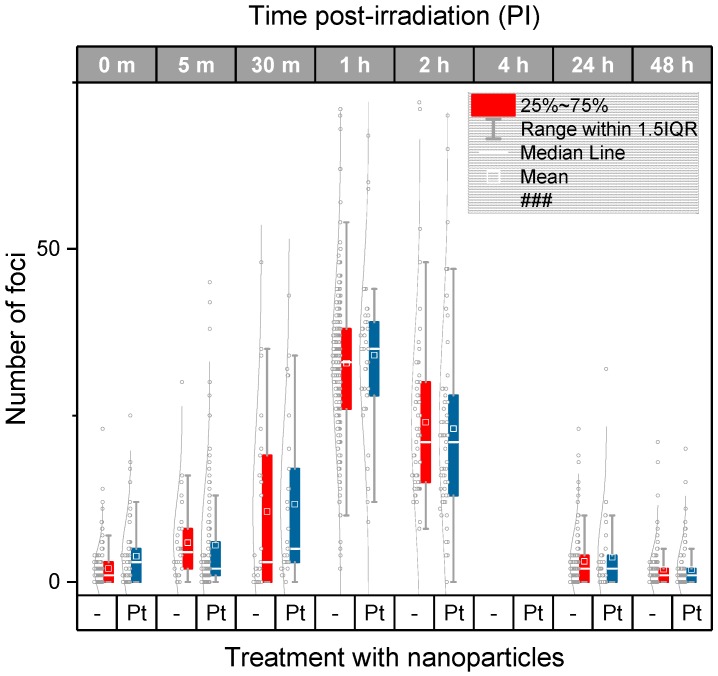
Automated analysis of the extent of γH2AX + 53BP1 focus (DSB) induction and repair kinetics compared for HeLa cells irradiated with 4 Gy of γ-rays in presence (0.5 mM for 6 h) or absence of 2.6 nm Pt-NPs. The average and median numbers of co-localized γH2AX+53BP1 foci (i.e., DSBs) per nucleus are shown for different periods of time PI, together with the focus number distributions in each cell population. The boxes include 50% of the values (25th to 75th percentile) centered on the median (the horizontal line through the box). The mean values are represented by the squares within the boxes. The outliers were identified according to the 1.5*IQR method (IQR = interquartile range). Pt—samples treated with platinum nanoparticles, m—the period of time after irradiation in minutes, 0 m—non-irradiated samples.

**Figure 6:**
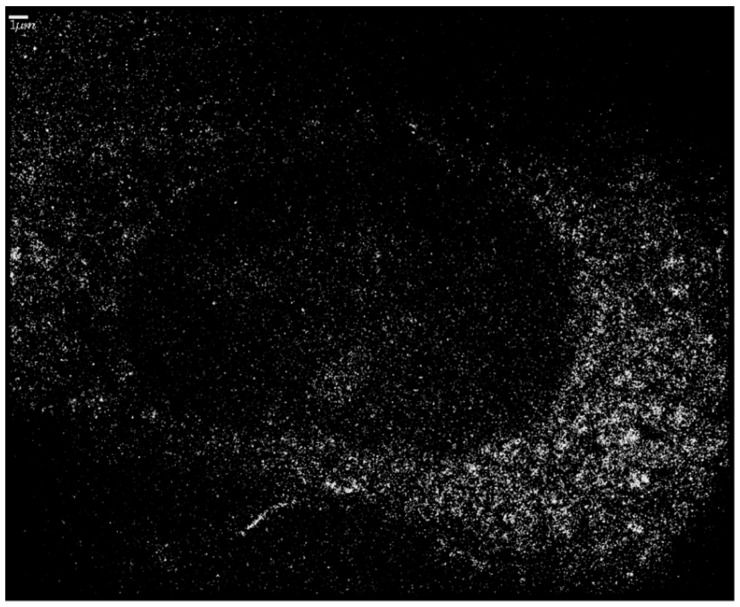
An illustrative Single Molecule Localization Microscopy (SMLM) image of an SkBr3 cell after uptake of 10 nm Au-NPs in the cytosol. The Au-NPs show a fluorescent blinking after laser illumination at 594 nm. Each point thus represents a single Au nanoparticle. Whereas the cytosol seems to be full of nanoparticles, the nucleus is empty. The points of low intensity seemingly covering the nucleus in the image either are the background or belong to out-of-focus image planes above or below the nucleus. Scale bar 1 µm.

Firstly, we analyzed potential negative influence of 2.6 nm Pt-NPs and 2.4 nm Au-NPs on the nuclear DNA of U87 and HeLa cells before irradiation. Cells were cultured with Pt-NPs or Au-NPs in the concentration of 0.5 mM for 6 h and potential induction of γH2AX/53BP1 (DSB) foci was studied as an indicator of nanoparticle-mediated genotoxicity. The repair foci have been present in both U87 and HeLa cell types already prior to incubation with nanoparticles and γH2AX foci mostly co-localized with 53BP1 protein. This observation points to a permanent existence of DSBs in U87 and HeLa cells, which is in accordance with their tumorous nature associated with genomic instability (Figure 2). U87 cells carried higher numbers of the foci than HeLa cells, with the mean values of 3.47 and 2.03, respectively. Figure 3, Figure 4 and Figure 5 (0 min post irradiation (PI) in all graphs) show that the average/median numbers of the foci per nucleus were almost identical (manual analysis, Figure 3) or increased slightly (automatic analysis, Figure 4 and Figure 5) after incubation of cells with Pt-NPs. The mean numbers of co-localized γH2AX/53BP1 foci per nucleus, provided by the automatic analyses, were 4.34 for U87 (Figure 3) and 3.88 HeLa cells (Figure 5). Such a differences, statistically significant though (U87: *p* = 0.010; HeLa: *p* = 0.003), are not supportive of biologically more relevant genotoxicity of the nanoparticles studied (2.6 nm Pt-NPs, and 2.4 nm Au-NPs; Figure 6), at least in terms of increased DNA fragmentation, consequently leading to genome rearrangements. Nevertheless, our studies limited to DSB induction cannot exclude a “milder” effect of nanoparticles on the DNA molecule, manifested for instance as oxidative base modifications. This kind of DNA damage may appear due to nanoparticle-mediated production of reactive oxygen species (ROS), which was frequently reported in the literature as the main cause of nanoparticle cytotoxicity. Moreover, especially in the context of what will follow, a negative potential of cytoplasmically localized nanoparticles may be preferentially or even exclusively targeted to the cytoplasmic structures. To summarize, our observations did not reveal more prominent genotoxicity of 2.6 nm platinum nanoparticles after short-term (6 h) incubation with U87 and HeLa cells, but more experiments are needed to comprehend potential cytotoxic effects of these nanoparticles in a more comprehensive way. Preliminary results seem to confirm this conclusion also for 2.4 nm Au-NPs.

### 2.3. DSB Induction and Repair in U87 Cancer Cells Treated or Not-Treated with Metal Nanoparticles Prior to Irradiation

After excluding the possibility that the studied 2.6 nm Pt-NPs and 2.4 nm Au-NPs markedly increase γH2AX/53BP1 focus (DSB) formation even by themselves, i.e., already in non-irradiated cells, we analyzed whether these nanoparticles can enhance DSB induction or affect DSB repair capacity of U87 and HeLa cells upon irradiation. The situation was compared for two γ-ray doses, 2 Gy and 4 Gy. We decided for a 2 Gy dose since this exposure is frequently used in clinical practice as a single fraction dose delivered to patients during a fractionated therapy. The higher dose of 4 Gy was applied in order to generate larger numbers of DSBs and explore differences between samples with better sensitivity (since the differences in DSB numbers per nucleus may be only small for low doses and therefore distinguishable from natural variability only with difficulty).

Figure 2 compares the γH2AX/53BP1 focus (DSB) formation and repair kinetics for U87 cells treated or not-treated with 2.6 nm Pt-NPs prior to irradiation with 4 Gy of γ-rays. Representative cell nuclei of both cell populations are displayed for different periods of time PI up to 48 h PI. Independently of the nanoparticle treatment, it is evident from Figure 2 that γH2AX foci are only incompletely formed in U87 cells early after irradiation (5–30 min PI) and also their co-localization with 53BP1 repair protein is very low. Correspondingly, the background signals (i.e., the proportions of γH2AX and especially 53BP1 molecules outside foci) are often high. A similar “picture” has also been reported for U87 cells exposed to heavy ions [62,63]. With ongoing time after irradiation, γH2AX and 53BP1 foci grow both in number and size and their mutual co-localization increases too. For both cell types (U87, and HeLa) and radiation doses (4 Gy, and 2 Gy), the number of co-localized γH2AX and 53BP1 foci reached the maximum between 30 min and 1 h PI. Later on, the number of foci started to decrease, while the size of foci gradually increased and the extent of co-localization between γH2AX and 53BP1 remained very high. Importantly (as quantified later), we did not observe any visual difference between nanoparticle-treated cells and their untreated counterparts with regard to the extent of DSB induction and repair kinetics. 

The quantitative results obtained for different ways of analysis (i.e., manual and automated) and two radiation doses (4 Gy and 2 Gy) are summarized in Figure 3, Figure 4 and Figure 5. Figure 3 compares the average/median numbers of γH2AX/53BP1 foci per nucleus together with the focus number distributions as gained by manual analysis for U87 cells exposed to 4 Gy of γ-rays in presence and absence of Pt-NPs, respectively. Except for two late time points PI (4 h and 24 h PI), all statistical characteristics (means, medians, and distributions) are almost identical for nanoparticle-treated and untreated cells.

The automated image analysis (Figure 4a) of the same cells that were previously evaluated manually provided much lower numbers of γH2AX/53BP1 foci compared to that in the manual analysis, especially at the early periods of time PI (up to 1 h PI). The maximum numbers of foci per nucleus were detected at 1 h PI in all samples, irrespective of the nanoparticle treatment and the way of analysis. During this period of time, about 50 foci per nucleus were counted manually while this value decreased to about 35 with the automated analysis. Taking into account previous reports showing that 1 Gy of γ-rays generates ~9–35 γH2AX foci per nucleus on average, depending on the cell type, the results of the manual analysis (mean = 12.5 foci/nucleus/Gy) can be considered as more realistic in terms of absolute numbers. A lower sensitivity of automatic analysis follows from the fact that computational parameters of focus scoring were set very strictly, just to detect only well-developed foci with an extensive overlap between γH2AX and 53BP1. The reason for this setting was to eliminate potential uncertainty with identification of small and/or immature foci since these foci could not be often easily separated from the background signal. Consistently, more prominent differences between the manual and automated analysis appeared at the shorter time points PI (up to 1 h PI), i.e., during the period of time when the representation of immature foci was high, especially in U87 cells. Under such conditions, automatic software analysis is still extremely difficult and manual analysis promises more precise results, especially in terms of counting the absolute focus numbers. On the other hand, computational analysis ensures detection of only precisely specified foci and therefore high reproducibility and objectivity of results, independently of the observer experience.

Another motivation to restrict the automated analysis selectively on well-developed foci followed from the question whether nanoparticles in irradiated cells may differently influence generation or repair of small and large γH2AX foci (the smaller foci were scored as DSBs by the manual analysis but not automated analysis). Except as described, both approaches provided very similar results despite of the different characters of manual and automated focus counting. Importantly, as for the manual analysis, the average numbers, medians, and distributions of γH2AX/53BP1 foci varied only inappreciably between U87 cells irradiated (4 Gy) with Pt-NPs present or absent. Very similar results for nanoparticle-treated and untreated samples were found also at 4 h PI and 24 h PI (Figure 4a), making the differences obtained for these time points by the manual analysis rather a deviation from otherwise tightly “overlapping” γH2AX/53BP1 focus profiles in time PI than a biologically relevant result.

For the lower radiation dose of 2 Gy of γ-rays (equivalent to a common single daily dose in fractionated radiotherapy), the same results as for the higher dose of 4 Gy were acquired (Figure 4b). Again, very similar numbers of γH2AX+53BP1 foci per nucleus were counted in irradiated U87 cells, irrespective of their incubation with Pt-NPs. Slightly higher mean numbers of γH2AX foci per nucleus were recognized in nanoparticle-treated cells compared to those in untreated ones only at 8 h and 24 h PI; however, comparable medians of the compared samples do not support existence of significant differences between Pt-NP-containing cells and controls even at these periods of time.

### 2.4. DSB Induction and Repair in HeLa Cancer Cells Treated or not-Treated with Metal Nanoparticles Prior to Irradiation

In the next step, we performed the same experiments as described in the previous chapter for U87 cells also with HeLa cervix carcinoma cells that differ from U87 cell by their origin and relatively lower radioresistance. Involvement of the two cell types into the study is important since the same nanoparticles may behave unequally in dependence of specific cell characteristics. Results for HeLa cells irradiated with 4 Gy of γ-rays in presence or absence of 2.6 nm Pt-NPs are compared in Figure 5. Though some differences in the extent of γH2AX/53BP1 foci formation and kinetics of their disappearance appeared between U87 and HeLa cells, 2.6 nm Pt-NPs added to HeLa cells cultures prior to irradiation (0.5 mM, 6 h-incubation) had no effect on DNA damage and repair, confirming thus our findings for U87 cells. Cell-type-specific extent of γH2AX/53BP1 foci induction and repair capacity might be attributed to different radiosensitivities of U87 and HeLa cells. In the present study, however, different levels of radioresistance and other characteristics of U87 and HeLa cells did not influence the processes initiated by nanoparticles in both non-irradiated and irradiated cells. Similarly, as described above for non-irradiated U87 cells incubated with 2.6 nm PT-NPs, addition of Pt-NPs by itself slightly increased γH2AX/53BP1 focus numbers per nucleus also in HeLa cells (i.e., without irradiation). This can be considered as a sign of potential genotoxicity of Pt-NPs, but biological relevance of this finding does not seem to be high.

### 2.5. SkBr3 Cancer Cells Treated or Not-Treated with Gold Nanoparticles Prior to Irradiation—Studying γH2AX Arrangement and Focus Formation by Single Molecule Localization Microscopy 

In the next step, we studied internal molecule arrangements and focus formation of γH2AX repair foci at the nanoscale by using SMLM [61]. These experiments can provide important new insights into the character of DSB damage generated by ionizing radiation in cells incubated or not incubated with metal nanoparticles. For these data, we assumed that the antibody tags against the H2AX phosphorylation sites represent the spatial topology of the foci. In the first approach, we therefore measured distance frequencies between labelling points and verified dose–efficiency curves on the point numbers in comparison to our recent approach [61].

**Figure 7:**
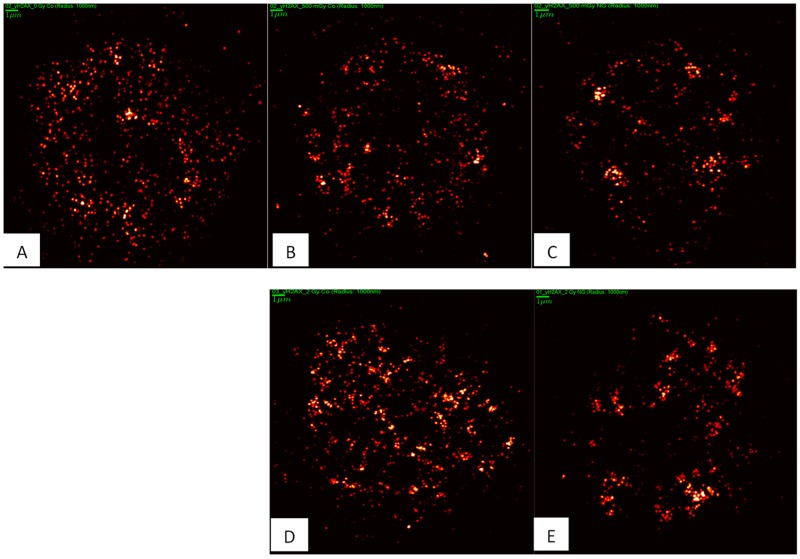
Illustrative SMLM next-neighbor density images comparing γH2AX labelling tag numbers and distributions in SkBr3 cells after irradiation (**B**,**D**) without particle incubation and after irradiation preceded by uptake of 10 nm Au-NPs into the cytosol (**C**,**E**). The intensity of the points represents the number of next neighbors in a 1000 nm radius environment. The control without any treatment (no NP-incubation, and no irradiation) is shown in (**A**), indicating some repair activity also in untreated cells. Images of irradiated cell were taken at 45 min PI. Scale bar: 1 µm.

SkBr3 cells were irradiated with 6 MeV X-rays at doses of 0, 0.5, 1, 2, or 4 Gy. For each dose, a specimen with and without 10 nm Au-NPs was irradiated. In order to ensure a maximum uptake and incorporation of these larger Au-NPs, incubation of cells was hold for 16 h prior to irradiation (Figure 6). Forty-five minutes after irradiation, the specimens were fixed and subjected to SMLM followed by software analysis of the H2AX labelling tags and their mutual distances. In Figure 7, typical next-neighbor density images are shown. In contrast to raw SMLM images showing just the positioning of fluorochromes with high precision (10–20 nm), these images encode the density of next neighbors in a 1000 nm environment by intensity. At a first glimpse, it seems that the cells with incorporated Au-NPs form more intensive foci, i.e., foci with more point signals than the cells that were irradiated with the same dose but without Au-NPs. In the case of the non-irradiated control, a random distribution may be supported by the visual impression, which contrasts with signal clustering in all irradiated cells.

**Figure 8:**
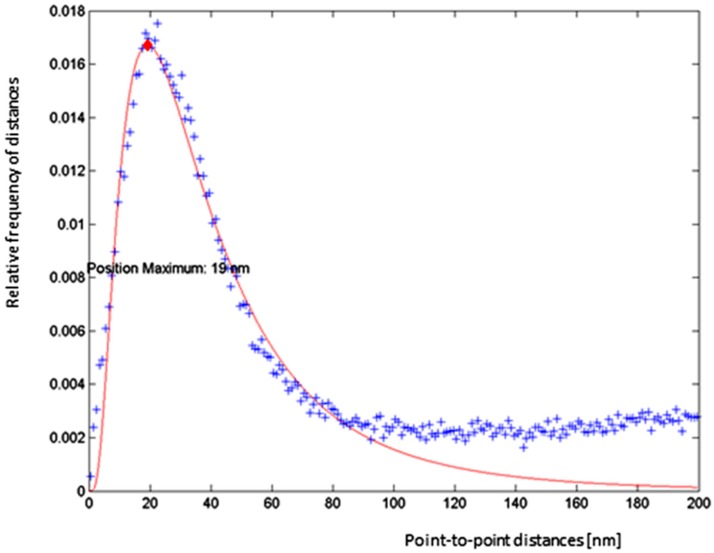
Example of a distance frequency distribution obtained for γH2AX labelling tags in an SkBr3 cell nucleus of the control (no NPs) specimen exposed to 0.5 Gy of X-rays. In all cases independent of the treatment, compatible distributions were obtained, indicating a characteristic non-random distance distribution. (blue crosses: number of measured distances; red diamond: peak maximum; red curve: fit curve)

**Figure 9:**
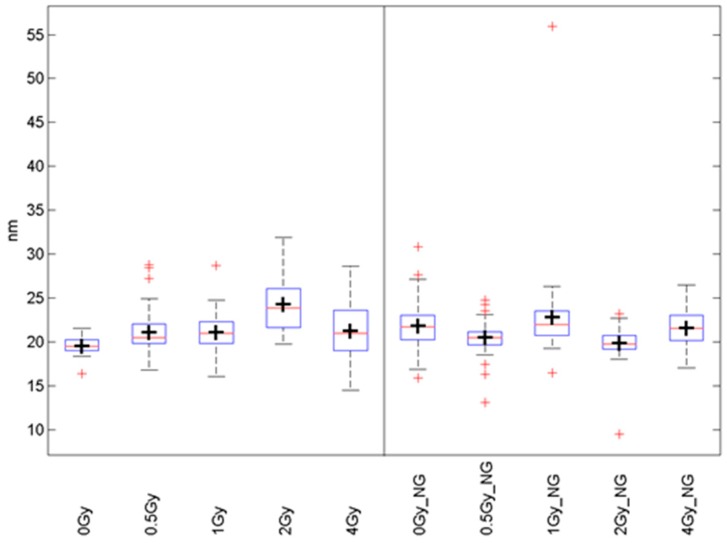
Boxplots of distance frequencies between γH2AX labelling tags in SkBr3 cell nuclei of irradiated specimens (left) and Au-NP-incorporated and irradiated specimens (NG, right). (black cross: mean value; red line: median value; red cross: outliner; blue box: first quartile; dashed line: standard deviation)

A more quantitative analysis based on Ripley’s K- and L-values [38] revealed a non-random distance distribution in all irradiated cell samples as it is shown for a case after 500 mGy radiation exposure without Au-NPs (Figure 8). This result indicates that, in all cases, clustering of γH2AX labelling tags can be expected. Therefore, we further studied the distance frequencies in order to find out whether the general γH2AX pattern is differing for the radiation doses and/or nanoparticle treatment conditions (Figure 9). In all irradiated cells, the average distance between γH2AX points was between 20 and 25 nm. Importantly, this γH2AX pattern did not change in specimens treated with Au-NPs. 

**Figure 10:**
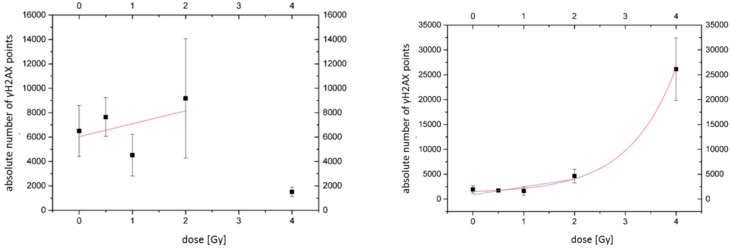
Dose–efficiency curves (number of γH2AX labelling tags vs. dose counted by SMLM). For the irradiated SkBr3 cell nuclei without Au-NP incorporation (left graph, a linear increase (red fit curve) can be observed at doses between 0 and 2 Gy. This was compatible to the blue linear fitting curve for irradiated SkBr3 cell nuclei with Au-NP incorporation (right graph. For the higher dose values, an exponential growth (red fit curve) or quadratic increase could be fitted to the values. (black square: mean value; error bar: standard deviation)

The numbers of γH2AX labelling tags can be used to determine the dependence of DNA damage extent on radiation dose and presence of nanoparticles [61]. Hence, we constructed preliminary dose–efficiency curves for X-ray doses up to 4 Gy and compared the numbers of γH2AX signal points in cells incubated or not incubated with Au-NPs (Figure 10). A slight linear increase in the number of γH2AX points was registered up to 2 Gy. In this dose interval, the curves were comparable for cells with and without Au-NP incorporation. Interestingly, a steep increase of the curve appeared between 2 Gy and 4 Gy after Au-NP incorporation, which was not observed in the control. In addition, the SMLM data indicate that the dose enhancement effects, as indicated by γH2AX signals, may be small, especially in dose ranges up to 2 Gy, which supports the data obtained above by ICM and foci counting. However, further experiments with other cell lines seem to be necessary for making the final conclusions.

In any case, we show here that the microscopic tools for nano-architecture analysis are available and adaptable to the challenges of NP-modified radiation treatment. The techniques of nano-probing and localization microscopy can be further improved by topological analyses of other repair foci (e.g., 53BP1 or Mre11) or analyses of chromatin conformation changes that may be induced by additional NP treatment. 

### 2.6. Compared Effects of Pt, Au and Gd Nanoparticles—Preliminary Results

**Figure 11:**
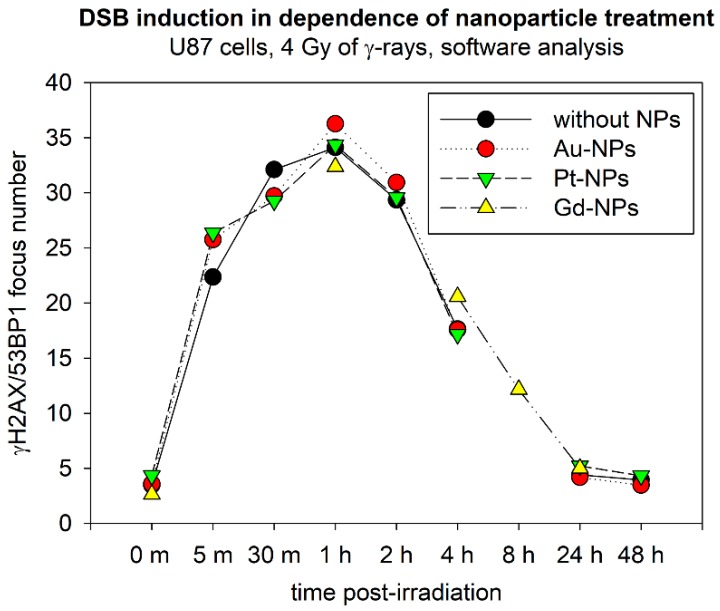
Comparison of γH2AX/53BP1 focus (DSB) formation and repair in U87 cells irradiated with 4 Gy of γ-rays in absence or presence of 2.6 nm Pt-NPs, 2.4 nm Au-NPs or 2.0 nm Gd-NPs. The results of an automated software analysis are shown as mean numbers of foci per nucleus measured at the indicated periods of time PI. Black circles—without NPs, green triangles—Pt-NPs (0.5 mM, 6 h-incubation), and red circles—Au-NPs (0.5 mM, 6 h-incubation; preliminary results). The data are also compared to our earlier results [21] for Gd-NPs (1 mM for 1 h, ^60^Co-irradiation, 4 Gy) (yellow triangles). X-axis: m = minutes, h = hours; 0 min = non-irradiated samples.

Finally, despite a preliminary character of the Au-NP data, we attempted here to compare DNA effects for three types of ultrafine (2–3 nm) metal nanoparticles composed of platinum, gold, and gadolinium, respectively. The size and incubation parameters were kept as similar as possible for all experiments to isolate only the effect of the nanoparticle material. The values for gadolinium(III) containing nanoparticles presented in Figure 11 were taken from our previous study performed with the same cells (U87) and under comparable experimental conditions [21]. As it is evident from Figure 11, the differences in DNA damage and repair between U87 cells exposed to 4 Gy of γ-rays after being or being not incubated with nanoparticles are quite small for all nanoparticles—platinum, gold, and gadolinium—studied. This means that 2.6 nm Pt, 2.4 nm Au and 2.0 nm Gd nanoparticles of given composition neither intensify DSB induction by ionizing radiation nor affect consequent repair of these lesions. 

**Figure 12:**
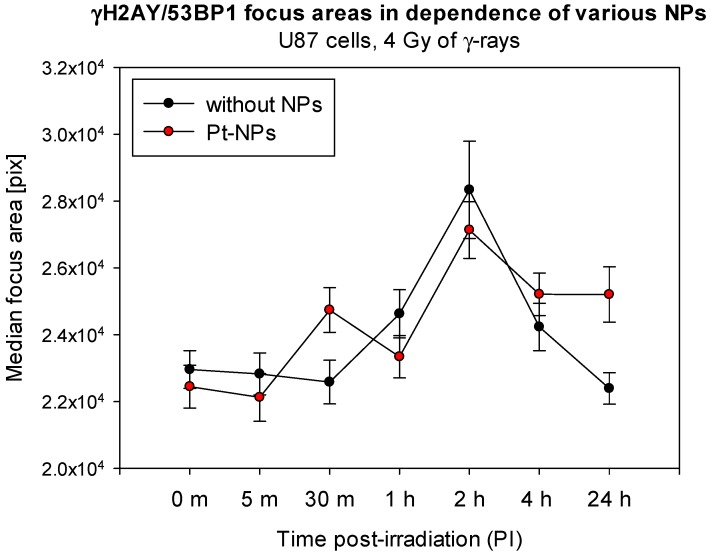
Comparison of γH2AX/53BP1 focus areas at different periods of time PI compared for U87 cells irradiated with 4 Gy of γ-rays in absence or presence of Pt-NPs. The results of an automated software analysis are shown as mean numbers of pixels per focus. Black circles—without NPs, and red circles—Pt-NPs (0.5 mM, 6 h-incubation; preliminary results). Error bars = standard error, m = minutes, pix = pixels, 0 m = non-irradiated samples.

Nevertheless, some indications can be recognized in our summarized data, eventually pointing to a delay in DSB repair, though the overall repair capacity of U87 cells has remained uninfluenced. Such a delay could be theoretically explained by a higher complexity of DSBs generated in presence of NPs. Therefore, as a rough estimation of DSB complexity, we quantified by Immune Fluorescence Microscopy) IFM the γH2AX focus areas for U87 cells irradiated (4 Gy) in presence or absence of Pt-NP nanoparticles. The results are presented in Figure 12. The curves for nanoparticle-treated cells and untreated controls seem to diverge starting with 4 h PI, indicating increased volumes of γH2AX foci in cells incubated with 2.6 nm Pt-NPs. Though these data are rather preliminary and experiments are needed for more nanoparticle types, well compatible results came also from SMLM nano-analyses, showing, compared to irradiated but untreated cells, more intensive γH2AX foci composed of more γH2AX signals in cells irradiated in presence of 10 nm Au-NPs. However, it remains difficult to explain why the complexity but not the extent of DSB damage increased in presence of NPs. Alternatively, cytoplasmically located NPs may enhance radiation damage to the cytoplasm. Consequent suboptimal condition of cells may indirectly decrease DSB repair. This could be supported by the observation that potential indications of a slower repair in nanoparticle-treated cells appeared only in later periods of time PI. However, it should be emphasized that, as a whole, our results rather support the no-difference scenario for nanoparticle-treated and untreated irradiated cells.

## 3. Discussion

The radiosensitizing effect of metal nanoparticles on tumor cells has been widely reported in the literature [22]. However, the mechanism or even multiple mechanisms of this potential radiotherapy improvement remains unknown. From the potential of physics [31], nanoparticles would have multiple benefits in cancer diagnosis and radiation treatment. They have been used as contrast agents [51] and locally for tumor damaging [24,64]. Functionalized nanoparticles can be used as vehicles for bio-molecules and drugs to infiltrate a tumor [23,48]. According to the cell-killing mechanism of ionizing radiation, which is based on DNA fragmentation through DSB induction, and the capability of metal nanoparticles to locally amplify the absorbed radiation dose at the microscale, a hypothesis on nanoparticle-mediated cell radiosensitization has been proposed and increased cell dying confirmed by colony-forming assays [42]. It has been well documented that irradiated nanoparticles, preferentially sequestered by tumor cells due to the so-called EPR effect and other effects, emit showers of secondary electrons that consequently increase water radiolysis around the sites of nanoparticle accumulations and damage important biomolecules, mainly the nuclear DNA. The Achilles’ heel of this otherwise very logical idea poses in a well-proved fact that while DNA is located in the cell nucleus, the nucleus is inaccessible even for nanoparticles of ultrafine dimensions (~2.5–10 nm) as used in the present study [53]. At the same time, the action radius of most secondary electrons kicked-off from cytoplasmically located nanoparticles is quite short [31]. Since some amounts of nanoparticles become concentrated around the cell nucleus or are specifically directed to the endoplasmic vesicles and reticulum, some secondary electrons may surely reach and damage the chromatin [23]. However, to what extent these rather rare acts of damage could contribute (increase) to cell killing remains a subject of debates. Moreover, the research on this topic is largely complicated by tremendous variability in the nanoparticle design (material, composition, size, shape, surface functionalization, etc.), cell-type-specific behavior and experimental conditions (type of radiation, radiation doses, nanoparticle concentrations and incubation times, etc.). Based on this situation, the aim of this article was to show by improved techniques of light microscopy whether cell radiosensitization by metal nanoparticles is correlated with an escalation of DNA damage expressed by the number of repair foci and/or affection of DNA damage repair capacity expressed by the maintenance of repair foci.

In the present work, we analyzed effects on DNA DSB induction and repair exerted by ultrafine nanoparticles composed of three different materials—2.6 nm Pt-NPs, 2.4 nm Au-NPs, 10 nm Au-NPs and 2.0 Gd-NPs—with an emphasis on result precision. All experiments were performed under the comparable conditions and in three different cancer cell lines (U87, HeLa and SkBr3) exposed to different doses (up to 4 Gy) of γ-rays or X-rays to reduce a potential bias of specific experimental conditions. U87 cells show very high radioresistance, which makes them an ideal candidate for a potential nanoparticle-enhanced radiotherapy. HeLa cells, on the other hand, are more radiosensitive and SkBr3 cells lie between HeLa cells and U87 cells. To monitor DSB induction and repair in a more comprehensive way, we quantified DSB numbers per nucleus at several time points PI, up to 24 h PI. This allowed us to compare the samples, not only the initial extent of DSB induction but also the kinetics and final efficiency of DSB repair. In addition, we were able to eliminate false differences between samples that could possibly appear if only one or two periods of time were followed. We used currently the most sensitive and accurate approach for DSB quantification—ICM of γH2AX and 53 BP1 repair foci in spatially (3D) fixed cells. γH2AX foci and 53 BP1 foci were evaluated in parallel and only co-localized foci of both DSB markers were considered as DSBs to further improve the quality and relevance of results. For the same reason, and to study small and larger γH2AX/53BP1 foci separately, we scored the foci both manually and automatically, by using novel software that is based on machine learning and has been purposefully developed and calibrated in our laboratory for the present analyses.

Taken all the ICM results together, we cannot confirm a significant effect of any nanoparticle studied (Pt, Au, and Gd) on the introduced number or repair efficiency of DSBs in irradiated cells. This conclusion holds true for both cell types (U87, and HeLa), radiation doses (4 Gy, and 2 Gy), and means of analysis used in the present study. Moreover, nano-scale SMLM studies on SkBr3 breast cancer cells with incorporated 10 nm Au-NPs also indicated that the spatial organization of γH2AX labelling tags seems not to be influenced by the presence of NPs in cells irradiated with different doses (0.5–4 Gy) of X-rays. The exception from this conclusion could be a slight delay of DSB repair in cells treated with 2.6 nm Pt and 2.4 nm Au nanoparticles in later (>4 h PI) time points PI. This difference in repair kinetics might be related to a larger size/higher intensity of γH2AX foci in nanoparticle-treated cells as observed for 2.6 nm Pt-NPs by ICM and for 10 nm Au-NPs by SMLM. However, it should be noted that the reported differences between nanoparticle-treated and untreated irradiated cells were only minor and non-systematic. We can therefore reasonably conclude that while nanoparticle-mediated radiosensitization has often been related to escalated DNA damage, the results presented here for ultrafine Pt and Au nanoparticles and also our earlier data for Gd nanoparticles [21] do not support this idea as a general mechanism responsible for the radiosensitizing phenomenon. Only for 10 nm Au-NPs and doses of 2 Gy or higher, some increase of γH2AX focus number was observed by SMLM, especially after nanoparticle modification for specific targeting to the ER [23], whereby this has only been observed for one cell line.

Our data suggest that there are at least some nanoparticles that increase cell killing upon irradiation [21,42] while they have none or a negligible effect on nuclear DNA break regions highlighted by H2AX phosphorylation sites. This confirms our intuition on the action mode of the radiosensitizing nanoparticles we developed. In other experiments (unpublished), we observed a great increase in the life span of animals bearing tumor (9L cell gliosarcoma in brain) or the inhibition of tumor growth (A375sc melanoma in flank) when the animals were treated by radiotherapy after intravenous (9L gliosarcoma) or intratumoral (melanoma) injection whereas the majority of the nanoparticles in the tumor were suspected to be outside the cells. Moreover, we also observed in preliminary experiments that the number of γH2AX is almost the same when irradiation is performed in presence or in absence of the radiosensitizing nanoparticles (unpublished results). Hence, these nanoparticles seem to sensitize cells to radiation through cytoplasmic effects that are independent of DNA damage and/or repair. While our findings do not exclude the possibility that some types of nanoparticles support radiation cell killing through the “classic” DNA damage-based mechanism, they open the door to exiting research of new mechanisms that could be dominant under some circumstances, as for instance chromatin topology-related effects and re-arrangements of compaction forms. Furthermore, accumulation of nanoparticles in endosomes and lysosomes as revealed in our earlier reports [21,55] could result in damage of these structures with important consequences. While lysosomes were originally thought only as cellular dustbins, recent studies involve lysosomes in important cell signaling pathways, eventually initiating apoptosis (see [32] and citations therein). In addition, even simple disruption of a larger amount of lysosomes due to their membrane damage by locally amplified radiation effects, mediated by intra-lysosomal nanoparticle accumulations, may result in massive leakage of lytic enzymes from these “suicide bags” [65,66] and extensive cytoplasmic damage. This can also initiate cell death. Indeed, the destabilization of lysosomes via lysosomal membrane permeabilization (LMP), leading to release of their aggressive content into the cytoplasm, is currently intensively studied as a potentially efficient way of therapeutic cell death triggering [56].

Cytoplasmically located nanoparticles may also influence organelles or structures which they do not co-localize with. For instance, increased production of ROS has been frequently reported in the literature as a main cause of nanoparticles’ cytotoxicity. Therefore, ROS generated by nanoparticles in extensive amounts upon irradiation may damage organelles located in close proximity to nanoparticle location sites, for instance mitochondria. Among other cytoplasmic targets, mitochondria are especially attractive since they are critical for cell survival (energy metabolism) and represent the only extracellular structures having their own DNA. Therefore, nanoparticle-mediated fragmentation of mitochondrial DNA may represent an elegant modification of the “classic” DNA damage-based hypothesis on cell radiosensitization by nanoparticles, returning this idea into the game. It should also be noted that ROS are effective signaling molecules with a strong potential to directly influence biochemical cellular pathways.

The Endoplasmic Reticulum (ER) may represent another target for nanoparticle effects. While the efficient functioning of the ER is essential for most cellular activities and survival, it may be under some modifications also invaded by nanoparticles [23]. Moreover, ER plays an important role in the response to oxidative stress-induced damage and is quite sensitive to ROS [67]. Hence, irradiated nanoparticles may exert cytotoxic effects on cells by modulating ER stress [67]. For instance, Ag-NPs resulted in cytotoxicity and cell death by apoptotic, which was associated with (secondary) DNA fragmentation [67]. This observation not only explains how nanoparticles may initiate cell death through disturbing functions of ER, but also stresses the importance of time when interpreting the nanoparticle-mediated DNA effects. In this light, it is possible that in some studies the nanoparticle-mediated effects on DNA can rather reflect this secondary apoptotic DNA fragmentation than primary enhancement of DSB induction by radiation. The mechanism, how ER stress can lead to apoptosis, has been described by [68]. A disruption of ER function leads to accumulation and aggregation of unfolded proteins accompanied with stress signaling. The stress signals are detected by transmembrane receptors, which in turn initiate the unfolded protein response (UPR) trying to restore normal ER functions. However, if the stress persists too long, apoptotic cell death ensues [68].

Altogether, we show that the radiosensitizing effect of at least some metal nanoparticles may rely on cytoplasmic processes rather than DNA damaging events. Based on the available literature, we also outline the way of how damage of the most relevant cytoplasmic structures may initiate cell death. Though we did not observe different responses to nanoparticles or irradiation in presence of nanoparticles for the two studied cell types (U87, and HeLa), we emphasize the necessity to analyze in detail each particular combination of nanoparticles and the cell type planned to be therapeutically targeted. This imperative follows from extensive controversies that are still present in the literature on the nanoparticle-mediated irradiation effects. For instance, Au-NPs induced apoptosis in MCF-7 and N87 cancer cell lines by disrupting lysosomes and mitochondria, but this effect did not appear in normal Chinese hamster ovary (CHO) and 293T cell lines. This observation further supports our conclusion that nanoparticle-mediated cell killing enhancement may be located in the cytoplasm, but more importantly gives a perspective of selective nanoparticle toxicity for tumor cells [64,69]. Interestingly, from the opposite point of view, some radio-protective chemicals (amifostine) protect normal cells from radiation effects but delay DSB repair in tumor cells [20]. 

The final question remains whether it is in principle a good or bad massage finding that nanoparticles damage the cells without affecting DNA. On the one hand, it could be beneficial since nanoparticles located outside the tumor will not increase the risk of genome damage and secondary malignancies induction in normal tissues surrounding the tumor. On the other hand, the radiosensitizing mechanism operating through DNA damage could be more efficient. A solution of this dilemma could be based on selective targeting of nanoparticles to specific genome sequences, like oncogenes, using appropriately designed oligo-nucleotides as being available for radio-emitters [70]. With techniques of COMBO-FISH [71,72] and PNA probe combinations [73], NPs may be transferred to cell nuclei and specifically addressed to given chromatin targets. This could be achieved by adding a nuclear localization signal (NLS) peptide motif and a specific PNA oligonucleotide probe to the surface of nanoparticles [74]. Using such sophisticated approaches of specific targeting of genome aberrations like multiple gene copies would open new aspects in tumor therapy. 

## 4. Materials and Methods

### 4.1. Cells and Cell Culturing

Three cancer cell types were studied in the present study: highly radioresistant U87 glioblastoma cancer cells, radioresistant SkBr3 breast cancer cells and relatively less radioresistant HeLa cervix cancer cells. U87 and HeLa cells were obtained from ATCC (American Type Culture Collection, Manassas, VA, USA). SkBr3 was commercially available and used for several SMLM studies in our laboratory. U87 and HeLa cells were grown in Dulbecco’s modified essential medium (Thermo Fisher Scientific, Waltham, MA, USA) supplemented with 10% heat-inactivated fetal calf serum (Thermo Fisher Scientific, Waltham, MA, USA), 100 U/mL penicillin (PAA), 100 μg/mL streptomycin (PAA), and 1% NEAA (Thermo Fisher Scientific, Waltham, MA, USA). Cell cultures were kept in T-25 cell flasks at 37 °C in a humidified atmosphere with 5% CO_2_.

For the experiments with SMLM, SkBr3 cells were prepared as described in detail elsewhere [72]. SkBr3 cells were grown in McCoy’s 5A cell medium, containing 10% fetal bovine serum (FBS) and 1% penicillin/streptomycin. Cells were cultivated and maintained at 37 °C in a humidified atmosphere at 95% air/5% CO_2_. Then, the cells were trypsinized and transferred to coverslips, put into six-well plates and further cultivated (about three passages, i.e., about 38 h) until 80% confluence.

### 4.2. Nanoparticles and Incubation of Cells with Nanoparticles

Platinum nanoparticles (Pt-PEG-17, referred to as Pt-NPs) were prepared as explained in the recently submitted French patent (FR 1900008). Briefly: Pt-NPs were synthetized by γ-ray water radiolysis of Pt containing salt and embedded with polyethylene glycol (PEG) to increase their biocompatibility. Pt-NPs were mainly spherical with an average platinum core diameter of 2.6 nm. Preliminary results were obtained also for gold nanoparticles (Au-NPs) which are composed of a Au core of 2.4 nm encapsulated by the dithiolated polyaminocarboxylate (DTDTPA) shell. For SkBr3 Au-NPs incorporation, 8 µL of 10 nm-sized gold particles (Aurion, Wageningen, The Netherlands) were added to the medium in each well 16 h prior to irradiation in order to obtain a maximum uptake in the cell cytoplasm via diffusion (Figure 6) [53,75]. In other experiments 2.6 nm Pt-NPs or 2.4 nm Au-NPs were added to the medium 6 h before irradiation at 0.5 mM concentration.

### 4.3. Cell Irradiation

Cells were irradiated in 6-well culture plates containing a culture medium and 2.0 × 10^4^–2.0 × 10^5^ cells per well. Consecutively, cells were exposed to 2 or 4 Gy of γ-rays (1 Gy/min), delivered by a ^137^Cs irradiator at room temperature (RT). During irradiation, the samples were kept in thermo-isolating boxes to prevent sample infection and temperature changes, and then immediately returned to the incubator (37 °C, 5% CO_2_) until taken for the experiment. For SMLM experiments, 10 nm Au-NPs were incubated 16 h before irradiation. Then, the cells were simultaneously exposed with and without Au-NPs using a 6 MeV Linac radiation source (Artiste, Siemens, Erlangen, Germany). The exposure doses of 0.5, 1, 2 and 4 Gy were obtained by changing the irradiation time at the same dose rate.

### 4.4. Immunodetection of γH2AX/53BP1 Foci and Double Strand Break Quantification

DNA DSBs were quantified in spatially (three-dimensionally = 3D) fixed cells by the means of high-resolution ICM detection of co-localized γH2AX and 53BP1 repair foci as described earlier [16,20]. Briefly, cells were fixed with 4% paraformaldehyde (10 min, at room temperature RT) prior to irradiation (0 min PI, non-irradiated controls) and in several time points PI covering a long (48 h) PI period (5 min, 30 min, 1 h, 2 h, 4 h, 8 h, 24 h and 48 h PI). Cells were permeabilized in 0.2% Triton X-100/PBS (15 min, RT) and immunoassayed with mouse antiphospho-H2AX (serine 139) (Merck, Darmstadt, Germany, cat. no.: 05-636) and rabbit anti-53BP1 (Cell Signaling Technology, Danvers, MA, USA, cat. no.: 4937) primary antibodies to simultaneously detect the γH2AX and 53BP1. Antiphospho-H2AX antibody was visualized with the secondary FITC-conjugated donkey anti-mouse antibody and anti-53BP1 antibody with Cy3-conjugated donkey anti-rabbit antibody (both Jackson Laboratory, West Grove, PA, USA, cat. no.: 715-095-150 and 711-165-152). Chromatin was counterstained with 1 μM TO-PRO-3 (Molecular Probes, Eugene, OR, USA) prepared in 2× saline sodium citrate (SSC). After brief washing in 2× SSC, Vectashield medium (Vector Laboratories, Burlington, Ontario, Canada) was used for sample mounting.

### 4.5. Fixation and Immunostaining of γH2AX for Single Molecule Localization Microscopy

45 min after irradiation, the cells were fixed in order to obtain an early response of the biological system to damage. The cells were washed in 1× Phosphate-Buffered Saline (PBS) with MgCl_2_ (0.901 mM)/CaCl_2_ (0.493 mM) and fixed in 3.7% formaldehyde (in 1× PBS + Mg/Ca; freshly prepared from paraformaldehyde) for 20 min at RT. After washing twice with 1× PBS + Mg/Ca, the cells were stored in 3.7% formaldehyde (in 1× PBS + Mg/Ca) at 4 °C. After 4-weeks storage, the formaldehyde was replaced by 1× PBS (+ 0.1% sodium azide). After removing the sodium azide from the coverslips, the cell membranes were permeabilized by 0.2% Triton-X100 three times for 5 min. After washing three times in 1× PBS (+ Mg/Ca), the cells were blocked in 2% bovine serum albumin (BSA) for half an hour and incubated in 100 μL of the primary antibody solution (mouse anti-phospho-histone H2A.X (Ser139) antibody; Merck Chemicals, Darmstadt, Germany; dilution: 1:500) at 37 °C for 18 h in a humidified chamber. Thereafter the coverslips with the cells were washed three times for 5 min with 1× PBS (+ Mg/Ca) to remove the remaining, unbound primary antibodies. Afterwards the secondary AlexaFluor 647 goat anti-mouse antibody was incubated in a humidified chamber at 37 °C for 30 min. Then, the cells were fixed in 2% formaldehyde (in 1× PBS (+ Mg/Ca) at 37 °C for 10 min. Finally, the cells were counterstained with 4′19,6-diamidin-2-phenylindol (DAPI; Sigma Aldrich, now Merck, Darmstadt, Germany) for 5 min in darkness and were, after washing twice with 1× PBS (+ Mg/Ca) for 5 min each, embedded in 20 μL ProLong Gold embedding medium (ThermoFisher Scientific, Waltham, MA, USA, ProLong Gold Antifade Mountant, P36930). The specimen was sealed and stored at 4 °C in complete darkness until SMLM application.

### 4.6. Confocal Microscopy

An automated high-resolution confocal fluorescence microscopic system Leica DM RXA [76,77,78], equipped with a CSU10a Nipkow disc (Yokogawa, Tokyo, Japan), an oil immersion Plan Fluotar objective (100×/NA1.3), a CoolSnap HQ CCD camera (Photometrix, Tucson, AZ, USA), and an Ar/Kr laser (Innova 70C Spectrum, Coherent, Santa Clara, CA, USA), was used for image acquisition [79,80]. About 40 individual confocal slices with 0.3 μm z-step increments across the nuclei were captured for each cell. Obtained images were analyzed using Acquiarium software [80] which enabled the three-dimensional reconstruction of images and inspection of individual γH2AX and 53BP1 foci in 3D space. Co-localized γH2AX/53BP1 foci were considered as DSBs to increase the precision of DSB detection, especially in the early-stage PI (with a higher background of signals) and also the probability that only unrepaired DSBs are still evaluated in later and very late periods of time PI.

### 4.7. Single Molecule Localization Microscopy

As described in detail elsewhere [61,81], the localization microscope used was equipped with four lasers to excite different fluorophores. The wavelength and the intensity of illumination were chosen by an acousto–optical tunable filter (AOTF). For our experiments, the 642 nm laser with 140 mW output power was used to stimulate the dye molecules to blink. A 100x/NA 1.46 oil immersion objective was used. The fluorescence of the specimen was separated from the illumination light by two quadband interference filters and was recorded by an EMCCD camera (Andor iXon Ultra 897, Belfast, UK). The EMCCD camera was operated at a gain of 100 and a series of 2000 up to 6000 image frames was recorded for each cell nucleus. Prior to the SMLM measurement, a widefield image was taken in the DAPI channel and the γH2AX channel with 10% laser intensity. Thereafter, the γH2AX image stack was recorded at 70% illumination intensity. Cells were chosen to have consistent size and form, a distinctive edge, a good staining signal-to-background ratio and a certain minimal distance to the next cell. The acquired data stacks were evaluated as described in detail in [61]. γH2AX labelling molecules were counted and distances between each point were determined. 

Data displayed in box graphs (Figure 9) show the frequency distributions of distances of γH2AX-labelling molecules. The boxes include 50% of the values (25th to 75th percentile) centered on the median (the horizontal line through the box). The mean values are represented by the squares within the boxes. The vertical lines begin at the 5th percentile and end at the 95th percentile.

### 4.8. Data Analysis and Statistical Evaluation after Confocal Microscopy

The SigmaPlot 14.0 (Systat Software Inc., San Jose, CA 95131 USA) and Origin 2018b (OriginLab Corporation, Northampton, MA 01060, USA) were used for data analysis and processing. The Mann–Whitney rank sum test was employed to compare γH2AX/53BP1 focus (DSB) numbers in untreated and nanoparticle-treated cells at all the particular periods of time PI. The results were considered as statistically significant at *p* < 0.05. The foci numbers were quantified both manually and automatically. In manual analysis, around 100 nuclei in each single experiment were blind-inspected (no information about the sample treatment) by eye by an experienced evaluator. For computational analysis, between 100 and 250 nuclei were scored. Because there is not a suitable tool fulfilling our demands on automatic γH2AX/53BP1 foci counting with our specific data, a custom program for fast and accurate foci counting, calibrated to our data, has been developed. The program works in a semi-automatic manner, where it allows for a visual inspection with the possibility to make quick manual adjustments and corrections, if necessary. The algorithm is composed of 3 steps—nucleus segmentation, foci segmentation and final foci classification, in order to eliminate false detections. Convolution Neural Network (with SegNet topology) was trained for robust nuclei segmentation, followed by splitting of touching nuclei with watershed transform applied on the distance transform of the segmented binary image. Inside a bounding box of each nucleus, the foci are segmented with a maximally stable extremal region detector, which is fast and invariant to image intensity values. The detector is set to high recall in order to obtain all possible foci for the classifier. Classification of true foci is done with Support Vector Machine classifiers on some extracted features (e.g., foci mean intensity and foci size). The program allows user to adjust the classifier bias value (to set classifier sensitivity), because the properties of foci are very heterogeneous between different samples and measurements. Besides the count of foci, it also allows exporting some other features for following analysis (cell size, foci sizes, foci intensities, etc.) (the full description of the software will be published separately). In Figure 3, Figure 4 and Figure 5, the data are displayed as box graphs also showing the distributions of DSBs foci per nucleus. The boxes include 50% of the values (25th to 75th percentile) centered on the median (the horizontal line through the box). The mean values are represented by the squares within the boxes. The vertical lines begin at the 5th percentile and end at the 95th percentile.

## 5. Conclusions

In the present study, we demonstrate that ultrafine (2–10 nm) platinum and gold nanoparticles do not escalate DNA damage or compromise DSB repair in irradiated tumor cells of different types. This confirms our recent findings for 2.0 nm gadolinium nanoparticles [21]. However, 10 nm Au-NPs may potentially influence the character of DNA damage at the nanoscale, as it was discovered by using SMLM [61,72,81]. Some indications in this sense have been obtained also by ICM for 2 nm Pt-NPs. While these findings are difficult to be interpreted in terms of biological relevance, contradictions still persist in the literature on the enhancement of nuclear DNA damage in cells irradiated in presence of metal nanoparticles. At the current stage of knowledge, it is reasonable to conclude that different mechanisms, involving an enhancement of DNA damage on the one side and cytoplasmic effects on the other side, participate in radiosensitization exerted by metal nanoparticles. More mechanisms probably contribute to the final radiosensitizing effect, involvement of which depends on the nanoparticle characteristics (material, size, composition, and surface functionalization), cell type and experimental conditions. Therefore, many questions on nanoparticle-mediated radiosensitization remain open, emphasizing the importance of more systematic future research. Methodologically, we demonstrate current possibilities and usefulness of the newly developed super-resolution microscopy technique (SMLM) that together with appropriate nano-probing technologies has a potential to shift our studies on DNA damage and repair to nanoscale dimensions. Mutual comparison of micro- and nano-scale results may provide a clue on many important processes taking part in cells and their molecular mechanisms.

## Abbreviations

DSB	double strand break
SMLM	single molecule localization microscopy
ICM	immunofluorescence confocal microscopy
Pt-NPs	platinum nanoparticles
Au-NPs	gold nanoparticles
PI	post-irradiation
EPR	enhanced permeability and retention
